# Efficacy of 24-week instability resistance training on cognitive function of older adults with probable mild cognitive impairment: A randomized, assessor-blinded, sham-controlled trial

**DOI:** 10.1016/j.jnha.2026.101030

**Published:** 2026-11-07

**Authors:** Dayane Tays da Silva, Bruno Remígio Cavalcante, Ryan Stanley Falck, Guilherme Moraes Balbim, Nádia Virginia Rodrigues Bezerra, Ana Beatriz Oliveira Ribeiro, Aline Batista Ribeiro Serqueira Rocha, Giordano Marcio Gatinho Bonuzzi, Carla Fernanda Ferreira Rodrigues Kursancew, Maria Joana Mesquita Cruz Barbosa de Carvalho, Rodrigo Cappato de Araújo, Mariana Ferreira de Souza

**Affiliations:** a Graduate Program in Physical Education (PPGEF), Universidade Federal do Vale do São Francisco (UNIVASF), Clinical Exercise Laboratory Research Group (LABEC), Petrolina, Pernambuco, Brazil; b Graduate Program in Rehabilitation and Functional Performance (PPGRDF), Universidade de Pernambuco (UPE), Petrolina, Pernambuco, Brazil; c Research Centre for Physical Activity, Health and Leisure (CIAFEL), Faculty of Sport (FADEUP), University of Porto, Porto, Portugal; d School of Biomedical Engineering, Faculty of Medicine, University of British Columbia, Vancouver, Canada; e Department of Physical Therapy, Faculty of Medicine, University of British Columbia, Vancouver, Canada; f Centre for Aging SMART at Vancouver Coastal Health Research Institute, Vancouver, Canada

**Keywords:** Exercise, Resistance training, Postural balance, Aged, Cognitive dysfunction

## Abstract

**Objective:**

To determine the effects of instability resistance training (IRT) on the cognitive function of older adults with probable mild cognitive impairment (MCI).

**Methods:**

This was assessor-blinded, randomized controlled trial which included 162 adults aged ≥65 years, physically independent and who had indicative of MCI. Participants were randomly allocated to either 24-week of twice-weekly IRT (n = 80) or active control group (n = 82). IRT group underwent resistance exercises on unstable surfaces (i.e., Balance Pad, Bosu® ball), prescribed as seven exercises in 3 sets with target zone ranging between 10–15 repetitions. The active control group performed non-structured (no progression neither complexity), low intensity functional and stretching/relaxation exercises. Cognitive outcomes Executive Function (Stroop Color Task; Digit Symbol Substitution Test; Trail Making Test Part A and B; Digit Span Memory Test; Verbal fluency test) and Memory were assessed at baseline, after 12- and 24-week. Linear mixed modeling, adjusted for sex, age, and baseline levels of each outcome, were used to determine treatment effects.

**Results:**

162 participants with a mean age of 70.4 ± 4.3 years and indicative of MCI (MoCA 20.1 ± 4.2) were included. At 24-week, the IRT group demonstrated significant improvements in executive function performance (Verbal Fluency – Letter A: +1.2 letters; 95% CI 0.2–2.2; p = 0.017; Digit Span Forward: +0.7 points; 95% CI 0.1–1.3; p = 0.010) compared with active control group. No significant between-group differences were observed in the remaining cognitive outcomes.

**Conclusion:**

Our findings revealed that 24-week IRT improved executive functioning domains among older adults with probable MCI.

**Trial Registration:**

RBR-9bv3dc9.

## Highlights

• Largest RCT to evaluate instability resistance training in older adults with MCI.
• A 24-week IRT program significantly improves executive functions in MCI.
• IRT may be a promising strategy for diversifying exercise prescriptions in older MCI.

## 1 Introduction

Mild cognitive impairment (MCI) is a key risk factor for dementia and perhaps the single most disabling condition in transition from mid-to-late life [1]. With few effective disease-modifying treatments available, prevention remains essential to protect cognitive health [2]. Non-pharmacological approaches such as exercise in the form of resistance training (RT) are increasingly popular strategies for promoting cognitive health [3]. Notably, RT improves executive functions (i.e., a set of higher-level thinking and planning abilities) and memory in older adults with preserved cognitive status as well as with indicatives/established cognitive impairment [4,5].

The cognitive benefits of RT have been elucidated through two complementary pathways: (i) an intensity pathway, whereby RT promotes neuroplasticity through metabolic stress and the release of neurotrophic factors (e.g., IGF-1, BDNF, and VEGF) [3,6]. MRI-based studies also suggest that RT might benefit brain structure and function (e.g., decreased burden of white matter lesions; region-specific increased gray matter volume; improved hemodynamics; functional connectivity within- and between brain networks) [3]; and (ii) a complexity pathway, in which novel and cognitively demanding motor tasks require sustained attention and recruit brain regions responsible for executive functions [7]. In this line, training modalities that simultaneously integrate physical intensity and motor complexity have emerged as promising strategies to enhance cognitive function. Instability resistance training (IRT) represents one such approach, involving moderate-intensity resistance exercises performed under unstable conditions [[8], [9], [10]]. It is known that IRT increases metabolic cost [11] and imposes greater coordinative and attentional demands [[12], [13], [14]], thereby promoting enhanced engagement of neural networks associated with cognition (e.g., executive functions) [15].

Previous studies have supported the view that IRT is effective to promote physical functioning (e.g., muscle strength, functional mobility and balance) and cognitive function among middle-aged and older adults [10]. However, considerable heterogeneity across studies results suggests the presence of bias and the necessity of further studies to properly reproduce the current evidence. For instance, many studies examining the effects of IRT on cognition have been predominantly short-term, have presented methodological issues (e.g., lack of blinding, small sample sizes, limited follow-up periods), and have mainly focused on cognitively healthy older adults [8,9,16]. Addressing this gap, our study aimed to assess the effects of 24-week IRT on cognitive function in older adults with probable MCI. We hypothesized that compared with a active control group, participants who underwent the IRT intervention would exhibit greater cognitive benefits.

## 2 Methods

### 2.1 Study design, setting, ethics and reporting statement

This was a single-center, parallel-group, randomized, assessor-blinded, randomized controlled trial. The study followed the principles of the Declaration of Helsinki and was approved by the Local Research Ethics Committee (CAAE No. 3 1043920.1.0000.5207; Protocol No. #67013617.4.0000.5196). Trial protocol was prospectively registered in the Brazilian Clinical Trials (ReBEC: https://ensaiosclinicos.gov.br/rg/RBR-9bv3dc9). All participants provided informed consent. The study reporting followed the Consolidated Standards of Reporting Trials (CONSORT) guidelines as extended to nonpharmacologic interventions [17,18].

### 2.2 Recruitment, participants and eligibility criteria

Community-dwelling older adults were recruited in two waves (February 16th and April 30th, 2022, and February 1 st and March 10th, 2023). Several strategies were implemented, including advertising on social media (e.g., Instagram and Facebook), local TV and radio, WhatsApp, and direct contact with community members (e.g., public markets, health services, and visits to churches and temples). We also sent invitations to previous participants of research and community programs within the local university. Participants were eligible if they met the following: a) aged 65 or older; b) physically independent (e.g., able to walk at least 10 m without the aid of devices such as a cane and walker); c) not engaged in structured physical exercise programs in the last three months; d) presented probable MCI, defined by the presence of at least one of the following criteria: (i) self-reported subjective cognitive complaints (e.g., Do you have any complaints of memory loss?); (ii) score lower than <26/30 points on the Montreal Cognitive Assessment Test (MoCA); or (iii) simultaneous presence of subjective cognitive complaints and <26/30 MoCA score [19]; e) no clinical restrictions or diseases that precludes safety throughout exercise classes; f) no diagnosis of cerebrovascular, neurological and/or psychiatric diseases; g) do not have complete proper cognitive assessments due visual and/or hearing impairments.

### 2.3 Randomization

Eligible participants were randomly assigned to the IRT or the active control group using a web-based randomization service (https://www.sealedenvelope.com/). This procedure was done remotely by an independent researcher at the completion of the entire baseline assessment. Unique identification numbers were generated to balance groups by age group (65–74 years vs ≥75 years), sex (Males/Females), and family members/social group (e.g., couples, siblings, or close friends). These procedures have been used previously in trials involving exercise for older people in anticipation of the potential age-related effects on training adaptations, greater prevalence of women as well as to minimize contamination and dropout rates between study groups [20,21].

### 2.4 Blinding

The cognitive function assessment was delivered by trained psychologists who were blinded to participants’ allocation. Over each study time-point assessment, they were asked to refrain from discussing their involvement or experience with interventions with assessors. Therapists who delivered the interventions were not blinded.

### 2.5 Sample size calculation

Our study was originally planned to determine the effects of IRT on falls incidence as the primary outcome, as stated in the trial registration, with cognitive function deemed a secondary outcome. To establish a reasonable number of participants for testing the hypothesis of superiority of IRT versus active control on cognition. We performed a sample size calculation based on previous studies examining the effects of traditional RT on cognitive function (assessed by Stroop Color Task) in community-dwelling older adults [6,16]. Assuming a two-sided alpha of 5%, a sample of 128 participants (64 per group) would provide a power of 0.80 to detect Cohen's d = 0.5. After accounting for 15% attrition and 1:1 allocation, a final sample of 150 patients (75 per group) was randomized to the IRT or active control group.

### 2.6 Interventions

Our training description followed the CERT guidelines [22]. The protocol prescription adhered to the recommendations of the American College of Sports Medicine [23], the National Strength and Conditioning Association [24] and the ICFSR guidelines [25] of RT for older adults. Participants underwent twice-weekly sessions (Tuesdays and Thursdays) with duration ranging from 50−60 min (per session) of IRT or active control group throughout 24-week. Both groups received the same motivational strategies to reinforce training engagement and controlling the social influence of behavioral intervention on cognitive outcomes [26]. These strategies included a friendly environment, music, social support, exercise protocol amendments (when needed) to accommodate musculoskeletal issues or mobility limitations (e.g., reduced range of motion, joint or muscle pain/discomfort).

#### 2.6.1 Instability resistance training (IRT)

Participants assigned to IRT underwent a structured training program, consisting of seven exercises using free-weights and bodyweight as follow: 1) wall ball squat; 2) chest press using free-weights; 3) dumbbells lunges; 4) rowing exercise using hanging straps (e.g., TRX); 5) pelvic elevation exercise; 6) abdominal exercise; and 7) standing calf raise. The prescription involved three fixed sets, each ranging from 10 to 15 submaximal repetitions. For the abdominal exercise, the prescription involved three sets from 15 to 30 submaximal repetitions or core/stabilization exercises with isometric contractions, with time varying from 10 to 30 s. The rest interval between sets and exercises ranged from 60 to 90 s. During the first 12 weeks of intervention, participants performed the traditional resistance training program without using unstable surfaces to ensure familiarization with the exercise protocol and to overcome significant deficits in muscle strength and balance. From weeks 13 to 24, participants progressively performed resistance exercises on unstable surfaces (e.g., BOSU®, foam pad, balance disc, Swiss ball, hanging straps). An illustration IRT exercises is presented in Fig. 1.

**Fig. 1:**
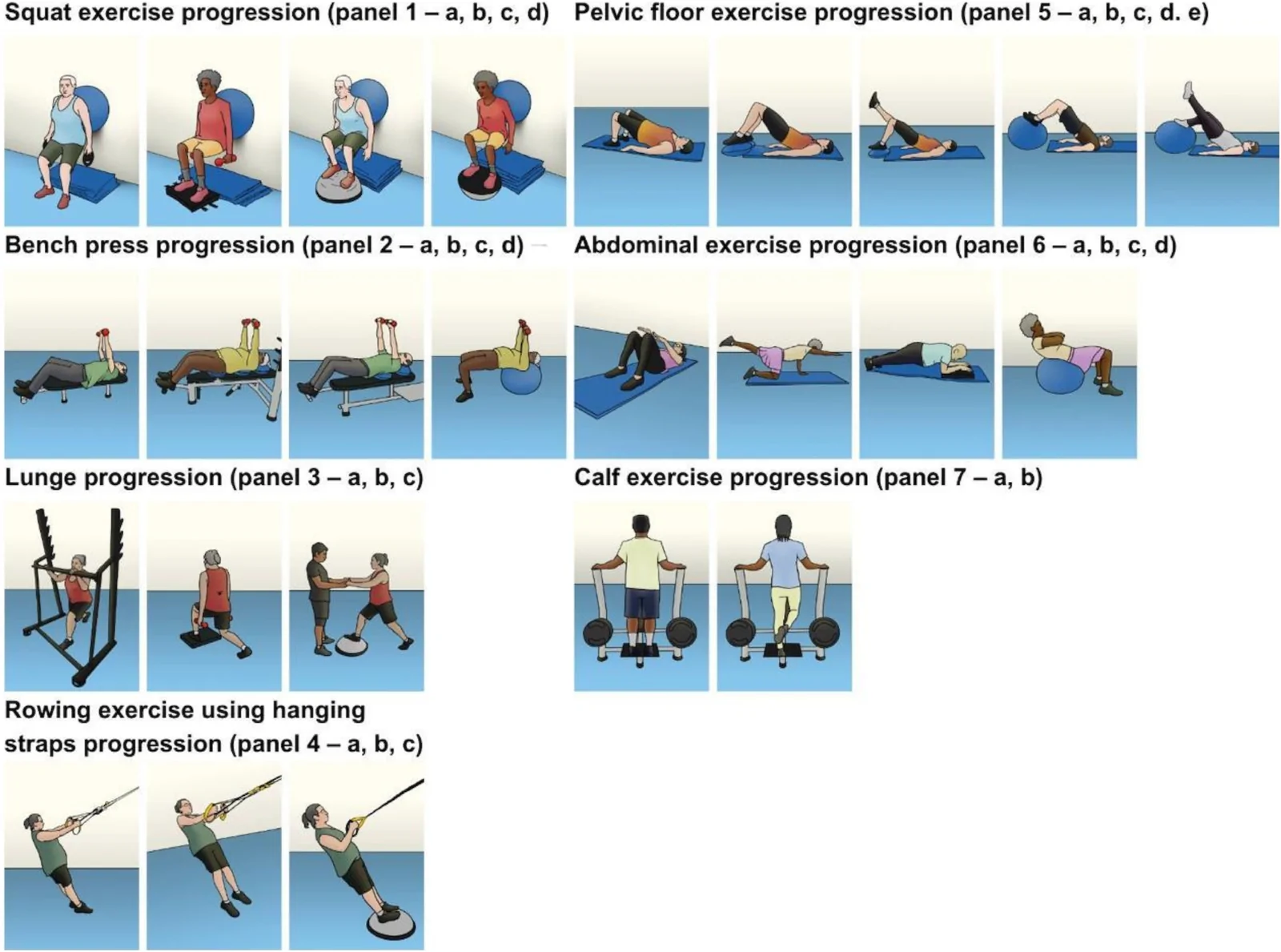
Progression of instability resistance training protocol.

We considered the following criteria to ensure exercise progression while taking IRT specificities into account: i) if a participant reached the upper limit of the target repetition zone (e.g., 15 repetitions) for three consecutive sessions with proper movement technique (e.g., adequate motor coordination and full range of motion), we increased the external load of exercises by 2%–10%; and ii) we increased the level of instability as the individual quickly improved their balance and/or muscle strength [23]. All sessions were supervised by trained instructors with one or two instructors per participant.

#### 2.6.2 Active control group

Participants assigned to the active control group participated in group-based sessions of unstructured, light-intensity functional, balance, and stretching/relaxation exercises, as well as recreational activities. Participants performed three fixed series of 10–15 repetitions, with a rest interval from 60 to 90 s. All sessions were supervised by trained instructors, with a ratio of one instructor to five participants. The rationale for this control group design was to minimize the influence of socializing effects of exercise training programs on cognitive function, as well as bias sources, such as the natural history of the disease, placebo effect, regression to the mean, Hawthorne effects, and recall bias [27,28].

### 2.7 Measurements

Data collection took place in the research laboratory of the Department of Physical Education at the Universidade Federal do Vale do São Francisco (Univasf). Assessments were conducted at baseline, after 12-and 24-week of intervention. The primary analysis focused on the outcomes measured at the end of the 24-week.

#### 2.7.1 Sociodemographic measures and health descriptors

Sample characterization data, including sociodemographic status (sex, age, weight, height, BMI, socioeconomic status, education and race) and health profile (presence of risk factors and associated diseases), were collected via telephone call during screening. Independence in basic and instrumental activities of daily living (Lawton-Brody scale) [29] and (Katz scale) [30], depressive symptoms (Geriatric Depression Scale — GDS-15) [31] and global cognitive function (Montreal Cognitive Assessment — MoCA) [19].

#### 2.7.2 Outcomes

We assessed specific domains of executive functions using the Stroop Color Task, Digit Symbol Substitution Test, Trail Making Test A and B, Digit Span Memory Test, semantic and phonological verbal fluency test. We also assessed logical memory throughout the Wechsler Memory Scale (WAIS-III).

##### 2.7.2.1 Specific domains of executive functions

The Stroop Color Task evaluate domains of selective attention and conflict resolution (ICC’s 0.71–0.79) [32,33]. In the Stroop Color Task, participants must verbally pronounce the presented color as quickly as possible. The test consists of three phases presenting different conditions: Phase 1) colored words, to mention the painted color; Phase 2) Neutral words in black color, to read what is written; Phase 3) Colored words, to identify the color of the ink and ignore the word itself. All phases were timed, the shorter the time to carry out the activity, the better the cognitive performance.

The Digit Symbol Substitution Test assesses processing speed, sustained attention and visual spatial abilities (ICC’s 0.79–0.97) [34]. Participants received a sequence of numbers with their corresponding symbols, and a paper containing only numbers and empty boxes to fill in. The Digit Symbol Substitution Test consists of filling in as many empty boxes as possible with the symbol corresponding to each number for seconds. The final score corresponds to the number of codes correctly, therefore, the higher the score, the better the cognitive performance.

The Trail Making Test (TMT) A and B assess processing speed and set shifting, respectively. The subtraction of TMT part B minus A evaluates cognitive flexibility (ICC’s 0.76–0.89) [35]. TMT A consists of connecting numbers in ascending order from 1 to 25. TMT B consists of connecting numbers and letters in an alternating, progressive manner (e.g., 1-A-2-B-3-C). TMT A and B were timed, the shorter the time, the better the cognitive performance.

The Digit Span Memory Test assess immediate auditory recall (ICC’s 0.71−0.88) [36]. In the Digit Span Memory Test, the evaluator read aloud random sequences of numbers, starting with three digits and ending with nine digits. Participants had to repeat each sequence exactly in the given order of numbers. It is divided into two parts: 1) forwards, the participant repeats the numerical sequence exactly as dictated by the evaluator; 2) backwards, the participant repeats the numerical sequence backwards. In both parts, the test ends when the participant cannot remember two consecutive sequences. The final score is based on the number of successful sequences, from 0 to 14 points (each part). The higher the score, the better the cognitive performance.

The semantic and phonological verbal fluency test was used to assess the language domain. The verbal fluency test consists of evaluating the number of words produced spontaneously, orally, for 60 s (per category). The semantic category, participants recalled words beginning with the letters F, A, and S, and the number of words produced in 60 s for each letter was recorded. And the phonological category sums the number of animal names. The final score is based on the number of words, the higher the score, the better the cognitive performance.

##### 2.7.2.2 Memory

The Wechsler Memory Scale (WAIS-III) [37] was used to assess immediate and delayed memory. The WAIS-III instructs the evaluator to tell two stories, one at a time. To assess immediate memory, the participant verbally recalls any information recalled at the end of each story. Delayed memory was assessed following a 30-minute break, wherein the participant verbalized any information remembered from the first and second stories. The final scores refer to “units of memories”, both immediate and delayed, therefore, the higher the score, the better the cognitive performance.

### 2.8 Retention and adherence rates

Retention was calculated as the number of participants who completed the post-assessments. Adherence to the intervention was assessed by session attendance, measured as the proportion of sessions completed out of the total sessions. The COVID-19 pandemic did not affect the conduct or timeline of the trial. No delays, protocol modifications, or interruptions related to COVID-19 were observed.

### 2.9 Statistical analysis

All analyses were conducted in R version 4.2.2. (R Project for Statistical Computing). Data management and visualization were performed using the *tidyverse* package collection. Linear mixed-effects models (LMM) were fitted using the *lme4* and *lmerTest* packages, while estimated marginal means and between-group contrasts were calculated using *emmeans*. Participants’ descriptive analysis was performed to summarize baseline characteristics. The effects of a 24-weeks IRT on cognitive function were examined using LMM with restricted maximum likelihood estimation. The model included random intercepts and fixed effects for time (baseline, 12-, and 24-week), group assignment (i.e., IRT, Sham), and their interaction (group × time). Each model was controlled for age (years), sex (male/female), education (years), and baseline outcome values were included as fixed-effect covariates. Unequal variance was allowed across time and groups. Estimated marginal means and between-group differences (IRT vs. active control) at 12-and 24-week follow-up were calculated. The overall α was set at .05 (2-sided). The statistical analysis followed intention-to-treat principles.

## 3 Results

A total of 463 participants were screened for eligibility; 301 were excluded for not meeting the eligibility criteria. Regarding eligibility criteria, among the 162 participants included in the study 7 participants (4.3%) were included based solely on subjective cognitive complaints, 37 (22.8%) based solely on MoCA scores <26, and 118 (72.8%) met both criteria. The one hundred and sixty-two participants were randomized into two groups (IRT = 80; active control = 82). The detailed flowchart is shown in Fig. 2. The dropout rate was 17.7% in IRT (14 of 79) and 15.8% in active control (13 of 82). In the final analysis, 78 of the 80 participants allocated to the IRT group were included, with two excluded due to a cancer diagnosis. Eighty-one of the 82 participants allocated to the active control group were included, with one participant excluded due to death.

**Fig. 2:**
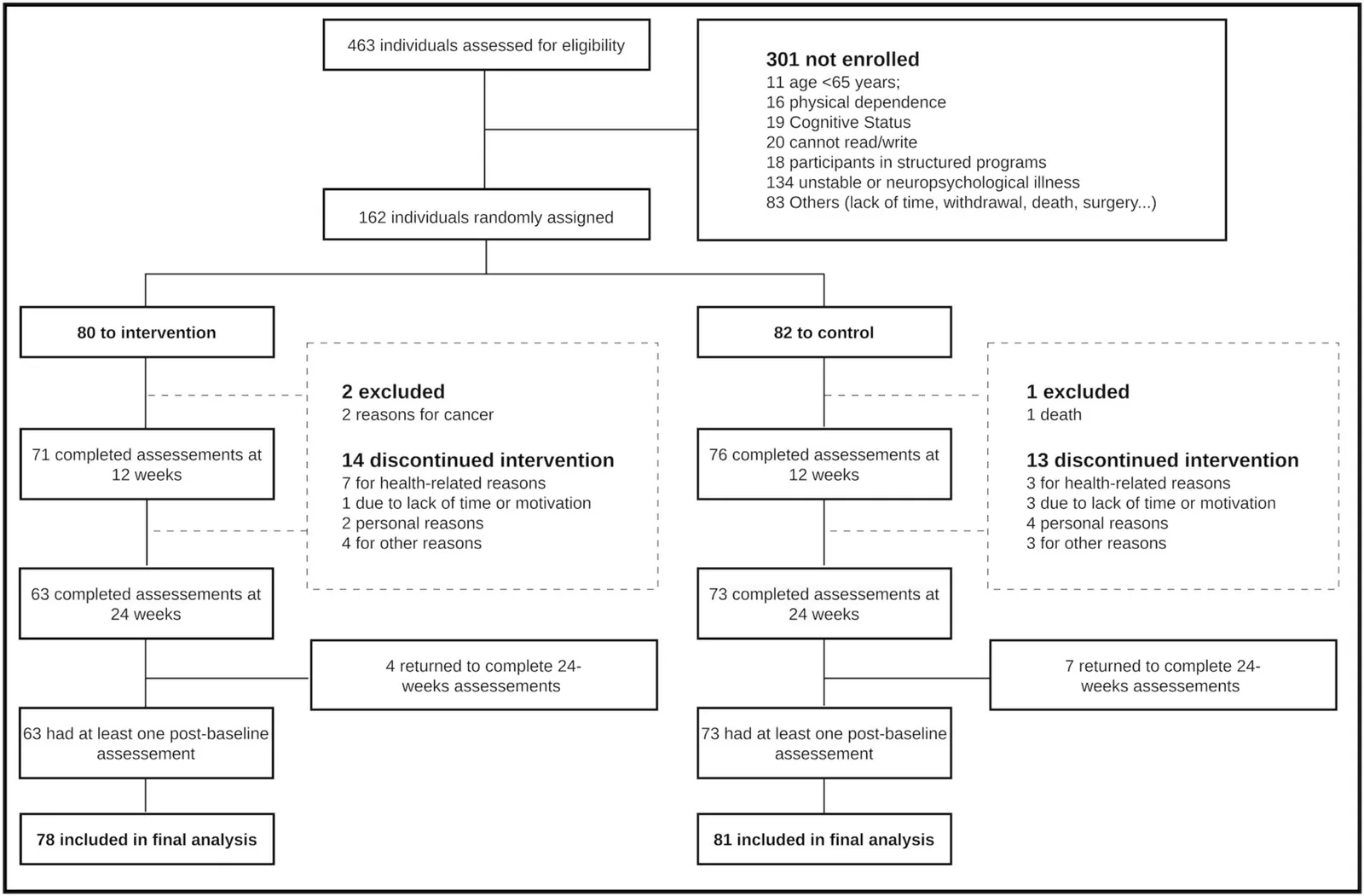
Flow diagram of the study.

The baseline characteristics of the participants are shown in Table 1. Most participants were female (75%), with a mean age of 70.4 ± 4.3 years and 11.4 ± 4.5 years of education. They were classified as physically independent in performing basic and instrumental activities of daily living. All participants completed assessments of global cognitive status and depressive symptoms, with mean scores of 20.1 ± 4.2 points on the MoCA and 3.24 ± 2.17 points on the Geriatric Depression Scale (GDS-15).

**Table 1:** General characteristics of participants.

Variables	Total (n = 162)	IRT (n = 78)	Active control (n = 81)
*Sociodemographic*			
Age, years	70.4 ± 4.3	70.6 ± 4.1	70.2 ± 4.6
Weight, kg	69.3 ± 14.9	69.8 ± 15.9	68.7 ± 14.0
Height, meters	1.58 ± 0.08	1.59 ± 0.08	1.58 ± 0.08
BMI, kg m^−2^	27.5 ± 5.1	27.5 ± 5.2	27.5 ± 5.0
Sex, n (% of females)	122 (75)	60 (75)	62 (76)
Race, n (% of non-white)	109 (75)	60 (75)	49 (60)
Income, number of wages*	2.8 ± 2.2	3.1 ± 2.8	2.5 ± 1.4
Education, years	11.4 ± 4.5	11.8 ± 4.3	11.0 ± 4.7

*Functional and cognitive status, behavioral aspects*			
IADL, score^a^	25.4 ± 2.8	24.9 ± 3.6)	25.8 ± 1.6
BADL, score^a^	17.8 ± 1.4	17.7 ± 2.0	17.9 ± 0.4
SPPB, score^a^	9.6 ± 1.4	9.5 ± 1.4	9.7 ± 1.4
MoCA, score^a,^**	20.1 ± 4.2	19.8 ± 4.1	20.3 ± 4.4
Falls history***, n (% yes)	52 (32)	24 (30.0)	28 (34)
Leisure time PA, n (% yes)	73 (45)	38 (47.5)	35 (43)

*Health status*			
Hypertension, n (% yes)	99 (61)	44 (55)	55 (67)
Diabetes, n (% yes)	35 (22)	18 (22)	17 (21)
Dyslipidemia, n (% yes)	48 (30)	27 (34)	21 (26)
Musculoskeletal disorders, n (% yes)	101 (62)	45 (56)	56 (68)
Low back pain, n (% yes)	79 (49)	42 (52)	37 (45)
Covid infection, n (% yes)****	46 (28)	21 (26)	25 (30)

*Abbreviations*: IRT- instability resistance training; BMI- Body mass index; IADL- Instrumental activities of daily living; BADL- Basic activities of daily living; SPPB- Short physical performance battery.

a Higher values represent better performance.

* Brazilian minimum wage corresponds to nearly U$265 (years of reference: 2022–2023).

** Based on Brazilian version of the Montreal Cognitive Assessment (MoCA).

*** History of falls in the previous year.

**** History of mild COVID-19 infection without hospital admission.

The effects of IRT on cognitive function over 12- and 24-week of intervention are presented in Table 2. The IRT group demonstrated a significant improvement in processing speed after 12-week compared with the active control group (TMT B: −14.2 s; 95% CI 0.8–27.7; p = 0.038). However, this between-group difference was not sustained at 24-week (TMT B: 0.2 s; 95% CI − 13.4–13.9; p = 0.966). After 24-week, participants in the IRT group exhibited significant improvements in working memory (Digit Span Forward: +0.7 points; 95% CI 0.1–1.3; p = 0.010) and in the language domain (Verbal Fluency – Letter A: +1.2 letters; 95% CI 0.2–2.2; p = 0.017) compared with the active control group. No significant between-group differences were observed in the remaining cognitive function domains.

**Table 2:** Efficacy of Instability Resistance Training on cognitive function among older adults with probable mild cognitive impairment.

Variables	Time-point: 12-week				Time-point: 24-week				Between-group differences 12-week*				Between-group differences 24-week*			
	IRT (n = 78)		active control (n = 81)		IRT (n = 78)		active control (n = 81)									
	Mean	SE	Mean	SE	Mean	SE	Mean	SE	EMM	LL	UL	p-Value	EMM	LL	UL	p-Value
Stroop 3–2, seconds^b^	74.1	3.5	74.2	3.5	77.8	3.6	71.1	3.4	−0.1	−9.3	9.0	0.979	6.8	−2.6	16.1	0.154
DSST, score^a^	22.8	0.7	23.9	0.7	23.9	0.7	24.3	0.7	−1.2	−2.9	0.6	0.196	−0.4	−2.2	1.4	0.671
TMT A, seconds^b^	93.7	4.8	86.6	4.8	95.0	5.0	92.1	4.7	7.1	−5.6	19.9	0.271	−2.9	−15.8	10.1	0.663
TMT B, seconds^b^	222.0	5.0	208.0	5.1	210.0	5.3	210.0	5.0	**14.2**	**0.8**	**27.7**	**0.038**	0.2	−13.4	13.9	0.966
TMT B–A, seconds^b^	129.0	6.6	122.0	6.8	118.0	7.0	116.0	6.6	6.5	−11.3	24.3	0.474	2.7	−15.5	20.8	0.772
Digit Span F, score^a^	5.8	0.2	5.9	0.2	5.8	0.2	5.1	0.2	−0.1	−0.7	0.5	0.849	**0.7**	**0.1**	**1.3**	**0.01**
Digit Span B, score^a^	4.1	0.2	4.2	0.2	4.2	0.2	4.0	0.2	−0.1	−0.6	0.5	0.528	0.2	−0.3	0.7	0.408
Digit Span F–B, score^b^	1.9	0.2	1.7	0.2	1.7	0.2	1.1	0.2	0.2	−0.4	0.8	0.480	0.6	−0.0	1.2	0.056
VF letter F, no. letters^a^	12.5	0.4	12.4	0.5	12.6	0.5	12.8	0.4	0.1	−1.1	1.3	0.872	−0.1	−1.3	1.1	0.855
VF letter A, no. letters^a^	10.0	0.4	10.4	0.4	11.1	0.4	9.9	0.4	−0.4	−1.4	0.6	0.412	**1.2**	**0.2**	**2.2**	**0.017**
VF letter S, no. letters^a^	9.5	0.4	10.2	0.4	10.4	0.4	10.2	0.4	−0.7	−1.7	0.3	0.179	0.2	−0.9	1.2	0.751
VF Animals, no. letters^a^	13.5	0.4	14.1	0.4	13.8	0.4	14.3	0.4	−0.6	−1.5	0.4	0.257	−0.5	−1.5	0.4	0.297
Immediate memory, score^a^	20.8	0.8	18.9	0.8	20.1	0.8	20.0	0.8	1.7	−0.4	4.0	0.108	0.7	−1.5	3.0	0.518
Delayed memory, score^a^	16.3	0.8	14.1	0.8	15.9	0.8	16.3	0.8	**2.2**	**0.1**	**4.2**	**0.036**	−0.3	−2.4	1.7	0.762

*Abbreviations*: IRT Instability Resistance Training; active control group; SE Standard Error; EMM estimated marginal mean differences; LL- 95% lower limit confidence interval; UL- 95% upper limit confidence interval; DSST Digit Symbol Substitution Test; TMT Trail Making Test; VF verbal fluency test.

Bold values indicate statistically significante differences (p < 0,05).

a Higher values represent better performance.

b Lower values represent better performance.

* Adjusted by baseline values of outcome, age (years), sex (F/M) and education (years).

With respect to logical memory, 12-week of IRT resulted in significant improvements in delayed recall (+2.2 points; 95% CI 0.1–4.2; p = 0.036) compared with the active control group. However, after 24-week, no significant between-group differences were detected for either immediate or delayed memory.

### 3.1 Adherence

The mean adherence of participants over the 24-week was 64.6% for the RTI and 62.5% for active control group, including those who withdrew during the study. Participants who missed a scheduled intervention session were contacted by telephone to identify the reason for absence and to encourage adherence. The criterion for contact was the absence from a scheduled session. Due to variability in attendance, the number of phone calls differed among participants; however, this procedure was applied consistently across both groups, ensuring that all participants received a phone call whenever a session was missed.

### 3.2 Adverse events monitoring

One adverse event was recorded. During a training session, a participant lost balance while performing rowing exercise using ranging straps (e.g., TRX). The event was classified as mild as it did not result in injury, did not require medical attention, and did not interrupt the training protocol.

## 4 Discussion

This was the largest randomized controlled trial (RCT) who examined the effects of 24-week IRT on cognitive function of older adults with probable MCI. Our findings revealed that cognitive-induced benefits of IRT might be domain-specific, with participants experiencing improvements in language and working memory. Conversely, we did not observe statistical differences between the IRT and active control groups in other cognitive tasks.

Although the mechanisms underlying the cognitive benefits of exercise are not fully established, the literature suggests that adaptive pathways vary with exercise modality, intensity, and participant characteristics. The most accepted hypotheses are biologically mediated mechanisms associated with resistance training, such as the release of neurotrophic factors (IGF-1 and BDNF) and the regulation of pro-inflammatory cytokines [3,6]. An umbrella review systematic reviews of RCTs indicates that physical exercise consistently promotes cognitive benefits across all age groups, including older adults regardless of initial cognitive status [38]. Although network meta-analyses RCTs rank RT as one of the most effective strategies for healthy older adults (SMD = 0.88) [39] and combined interventions for those with MCI (SMD = 0.72) [40], subgroup analysis by Singh et al. (2025) reveals that modalities integrating movement with cognitively challenging tasks (such as exergames) show superior effect magnitudes in cognition (SMD = 0.61) and memory (SMD = 0.58). This advantage may be attributed to the synergistic activation of multiple neurobiological pathways stimulated by the demand for real-time mental processing [40,41].

This heightened attentional demand in IRT is supported by physiological evidence of neuromodulatory alert-system activation. A recent study in healthy young adults, indicates that performing exercises on unstable surfaces elicits greater pupil dilation compared with exercise on stable ground; this represents a physiological marker of locus coeruleus activation and the subsequent release of norepinephrine, which influences attention and arousal states [15]. Additionally, the literature suggests that this exercise model is related to competition for limited attentional resources, as complex motor tasks require the simultaneous engagement of multiple cognitive processes [14]. These theories help explain the large effect sizes for IRT on cognitive function reported in previous systematic review (*g* = 0.80; 95% CI 0.03–1.19) [10]. Our findings showed that, compared with active control, IRT group improved 1.2 words (95% CI: 0.2–2.2) in verbal fluency and a 0.7-point increase (95% CI: 0.1–1.3) in Digit Span tasks. Although these values indicate a positive direction in language, attention, and working memory, interpreting the clinical magnitude remains complex due to the absence of Minimal Clinically Important Difference (MCID) parameters specific to these tests. This gap highlights the need for advances in the psychometric properties of neuropsychological measures applied within the exercise context, enabling clearer interpretation of their true clinical impact.

The divergences observed in the literature regarding the effectiveness of IRT appear to be explained by intervention duration and the cognitive profile of the sample. Short-term interventions (10-week) have heterogeneous results. For example, Eckardt (2020) and Claußen (2025) demonstrated significant improvements in inhibitory control, processing speed, and working memory in cognitively unimpaired individuals when compared with other exercise modalities [8,9]. In contrast, Cavalcante (2020) observed that 12-week of IRT did not improve global cognitive function compared with a health education control group among older adults with cognitive complaints [16]. This disparity suggests that in populations exhibiting cognitive decline, short-duration protocols may be insufficient to detect changes in cognitive domains, and that greater exposure to exercise, such as the 24-week intervention adopted in the present study, may be necessary for benefits in language, attention, and working memory to emerge.

In this study, we did not observe statistically significant differences in logical memory between the IRT and active control groups. One plausible hypothesis for this finding may be attributed to the methodological rigor of the control group adopted. Unlike previous clinical trials that used usual-care control groups, the use of a active comparator allowed the isolation of the intervention's true therapeutic benefit. By mimicking the structure of the training sessions without load progression or instability challenges, we controlled for critical confounding variables such as the Hawthorne effect and memory bias [28]. Although the magnitude of the effects may appear modest compared with studies using inactive controls, our findings provide a more precise estimate of IRT's impact. Therefore, these data reinforce the premise that instability acts selectively, emerging as an important factor in optimizing some specific domains of executive functions but not necessarily in improving memory in older adults with cognitive impairment.

A major strength is that we conducted this RCT with a large sample, thereby enhancing both internal and external validity. The inclusion of an active comparator group helped control for attention-related biases and potential confounding factors. Outcome assessors were blinded at all three assessment time points, minimizing evaluator-related variability. The intervention was delivered under consistent and qualified supervision, further strengthening methodological rigor and protocol fidelity. However, some limitations should be acknowledged. We were unable to determine the specific subtype or severity of cognitive impairment, and the sample may therefore have included participants with heterogeneous cognitive profiles; the findings may not be generalizable to populations with different clinical characteristics or health profiles.

## 5 Conclusion

We conclude that 24-week of IRT improves executive functioning of older adults with probable MCI. These findings reinforce the value of IRT as a viable exercise modality for mitigating cognitive decline in this population and encourage healthcare professionals to consider its incorporation into training protocols targeting older adults with indicative signs of MCI.

## CRediT authorship contribution statement

M.F.S contributed to the conception and design of the study. D.T.S, N.V.R.B, A.B.O.R, A.B.R.C.R supervising the training programs and C.F.F.R trained the evaluation team. B.R.C analyzed the data. D.T.S interpreted the data, wrote the first draft of the manuscript and prepared Fig. 2, Tables Table 11 and Table 22. All authors contributed to manuscript revision, read, and approved the submitted version.

## Ethics approval

Ethics approval was obtained from Universidade de Pernambuco (UPE), Petrolina, Pernambuco, Brazil (CAAE No. 3 1043920.1.0000.5207; Protocol No. #67013617.4.0000.5196).

## Patient and public involvement

Patients or the public were not involved in the design, or conduct, or reporting, or dissemination plans of our research.

## Provenance and peer review

Not commissioned; externally peer reviewed.

## Declaration of Generative AI and AI-assisted technologies in the writing process

The authors declare that no generative AI or AI-assisted technologies were used in the writing, editing, or preparation of this manuscript, nor in the creation or modification of any figures, images, or illustrations.

## Funding

Mariana Ferreira de Souza holds grants from the Fundação de Amparo à Ciência e Tecnologia de Pernambuco (FACEPE) (APQ-0926-4.09/21) and the Conselho Nacional de Desenvolvimento Científico e Tecnológico (CNPq) (427517/2018-4 and 448113/2025-2). Bruno Remigio Cavalcante holds a grant from CNPq (447978/2025-0).

## Data availability

De-identified participant data may be shared upon reasonable request to the corresponding author, subject to ethical approval.

## Declaration of competing interest

The authors declare the following financial interests/personal relationships which may be considered as potential competing interests:

Mariana Ferreira de Souza reports financial support was provided by Foundation for Support of Science and Technology of Pernambuco State. Mariana Ferreira de Souza reports financial support was provided by National Council for Scientific and Technological Development. If there are other authors, they declare that they have no known competing financial interests or personal relationships that could have appeared to influence the work reported in this paper.
